# Bradycardia-induced Torsade de Pointes – An arrhythmia Less Understood

**Published:** 2010-10-31

**Authors:** Narayanan Namboodiri

**Affiliations:** Sree Chitra Tirunal Institute for Medical Sciences and Technology, Trivandrum, Kerala, India

**Keywords:** Torsades de Pointes, Bradycardia, Atrioventricular block

Torsade de pointes (TdP) denotes a polymorphic ventricular tachycardia that is associated with prolonged QT interval at baseline, and is potentially life-threatening. Though it is well established that the genesis of TdP is closely linked to the transmural dispersion of repolarisation (TDR), the cellular and electrophysiological milieu predisposing to torsadogenesis in many conditions is not completely understood [1].

Significantly increased TDR at contiguous myocardial sites functions as a re-entrant substrate to initiate and maintain a TdP episode. Increased TDR in myocardium is represented by the electrocardiographic finding of prolonged QT interval. More specifically, the descending limb of the T wave represents TDR and this phase is considered to be a vulnerable period for torsadogenesis [2]. When a premature beat occurs in this period, the arrhythmia is initiated and perpetuated due to phase 2 re-entry or phase 2 early after depolarization.

Bradycardia is known to be one of the major factors predisposing to TdP. The inverse relationship between heart rate and repolarization time primarily accounts for bradycardia-induced QT prolongation. In addition, bradycardia increases the torsadogenicity of drugs that block I_Kr_ because these drugs block K^+^ channels in a reverse-use-dependent manner [3]. TdP has been described in many conditions with bradycardia like atrioventricular (AV) block, drugs, vagotonia, hypothyroidism etc [4-7]. However, patients with chronic AV block may have mechanisms other than the magnitude of bradycardia. Pause-dependent TdP has been recognised as an important complication of chronic AV block for long [8].  Studies have noted that patients with heart block have a significantly longer QT interval than those with sinus bradycardia even at comparable heart rates [9].  This relative QT prolongation may be one of the major reasons why TdP is more commonly observed in chronic AV block than in sinus bradycardia. However, the cellular mechanisms accounting for prolongation of repolarisation in them may be varied. Early after-depolarisations may develop in these patients through prolongation of the action potential duration secondary to both bradycardia-dependent depression of electrogenic Na^+^ pumping and more complete inactivation of I_K_ [10]. In addition, low ventricular rates are associated with submaximal activation of I_TO_, which shifts plateau of the action potential to a voltage levels in which the Ca^2+^ window current availability is increased [11]. Fast heart rates tend to oppose these actions, preventing early after-depolarisations and TdP.

In this issue of the journal, Yiginer G et al report a retrospective analysis of 64 patients with chronic AV block for occurrence of TdP and its predictors [12]. The three patients who developed TdP in this group were females, had more advanced age, and were in bradycardia for longer duration. The gender-specific preponderance in females to develop drug-induced TdP when treated with antiarrhythmic drugs or during spontaneous bradyarrhythmias is already known [13]. Elderly persons are more likely to have more co-morbid conditions like hypertension, coronary artery disease and heart failure, and these co-morbidities are known to cause down regulation of potassium channels [14]. Duration of exposure to bradycardia is another factor predisposing to TdP according to the authors. Concomitant to the duration of AV block, the likelihood of slower and irregular ventricular rhythms, which can result in ‘long-short’ sequence, may also increase. Chronicity of bradycardia might result in many alterations in myocardial channel functions also [15]. These alternate mechanisms also may contribute to an increase in the degree of the dispersion of refractory periods and facilitate the development of extrasystoles, thus playing a major role in producing TdP, apart from the magnitude of bradycardia, in patients with chronic AV block.

The authors identified that T wave notching on ECG having a predictive value for TdP, besides QT prolongation. All 3 cases who developed TdP had notched T waves in the ECG on the occurrence day of TdP, and in them, Tpeak-Tend were longer than 85 ms. This is concordant with the previous observations in similar group of patients. Bozkaya et al noted the presence of prolonged QTc/JTc intervals, pathologic U wave and T-U complex, prolonged Tpeak-Tend interval, and LQT2-like QT morphology as the predictors of ventricular arrhythmias during chronic AV block [16]. In another retrospective case-control study, where genetic testing was done in patients with TdP in the setting of complete AV block, the terminal phase of repolarization (Tpeak to Tend) was identified as a marker of an underlying cardiac ion channel abnormality. In this study, 4/11 (36%) patients with complete AV block and TdP had a genetic mutation identified involving ion channels responsible for cardiac repolarization involving hERG (n=3) and SCN5A (n=1).  Authors proposed TdP in the setting of complete AV block to represent the subset of patients with underlying genetic predisposition to reduced repolarization reserve [17].

This study conveys a message that a subgroup of patients with chronic AV block are at higher risk to develop life-threatening ventricular arrhythmias. Presently, any of the predisposing factors identified by the authors - age, gender or duration of heart block- are not given added importance while recommending pacemaker implantation in patients with chronic AV block.  In real world scenario, large prospective randomised studies in patients with chronic AV block to assess the unintervened natural history of varying subgroups may not be possible because of multitude of reasons. Hence the recommendations are often supported only by evidence from retrospective observations. It is interesting to note that not a single recommendation (Class I-III) in the current ACC/AHA guidelines for pacing in bradycardia is backed up by level of evidence A [18]. In contrast, for both biventricular pacing and cardioverter-defebrillators, the benefits of implantation are overwhelmingly evidence-based. The reasons for this apparent paradox could be multifactorial. Pacemaker is clearly life saving at least in a selected population and may not need further evidence. Furthermore, only a few patients with unintervened natural history may be available to assess the long term outcome. These limitations leave a few clinical scenarios like asymptomatic chronic AV block with narrow QRS escape rhythm, especially while noted for the first time in adults, with limited evidence in favour of clear benefit with pacemaker implantation. Still, the present guidelines favour implantation as reasonable for persistent third-degree AV block with an escape rate greater than 40 bpm in asymptomatic adult patients without cardiomegaly (Indication: Class IIA, Level of Evidence: C). Interestingly, the recommendations do not consider QT prolongation as an indication for pacemaker implantation in these patients. Indeed, subsequent studies would be required to establish the incremental role of the high-risk predictors identified by Yiginer et al in further risk stratification of chronic AV block.

## References

[R1] Shimizu W (1999). Cellular basis for long QT, transmural dispersion of repolarization, and torsade de pointes in the long QT syndrome. J Electrocardiol.

[R2] Yan GX (2003). Ventricular repolarization components on the electrocardiogram: cellular basis and clinical significance. Journal of the American College of Cardiology.

[R3] Hondeghem L (1990). Class III antiarrhythmic agents have a lot of potential but a long way to go. Reduced effectiveness and dangers of reverse use dependence. Circulation.

[R4] Kurita T (1992). Bradycardia-induced abnormal QT prolongation in patients with complete atrioventricular block with torsades de pointes. Am J Cardiol.

[R5] Gupta A (2007). Current concepts in the mechanisms and management of drug-induced QT prolongation and torsade de pointes. American heart journal.

[R6] Farkas A (2008). Importance of vagally mediated bradycardia for the induction of torsade de pointes in an in vivo model. Br J Pharmacol.

[R7] Schenck JB (2006). Severe primary hypothyroidism manifesting with torsades de pointes. Am J Med Sci.

[R8] Locati EH (1995). Spontaneous sequences of onset of torsade de pointes in patients with acquired prolonged repolarization: quantitative analysis of Holter recordings. J Am Coll Cardiol.

[R9] Chawala P (2008). Pause dependent torsade de pointes - does the cause of bradycardia matter?. Circulation.

[R10] Antzelevitch C (1994). Clinical relevance of cardiac arrhythmias generated by afterdepolarizations. Role of M cells in the generation of U waves, triggered activity and torsade de pointes. J Am Coll Cardiol.

[R11] January CT (1992). Cellular mechanisms of early afterdepolarizations. Ann N Y Acad Sci.

[R12] Yiginer O (2010). Current concepts in the mechanisms and management of drug-induced QT prolongation and torsade de pointes. Indian Pacing Electrophysiol.

[R13] Kawasaki R (1995). Increased propensity of women to develop torsades de pointes during complete heart block. Journal of cardiovascular electrophysiology.

[R14] Tsuji Y (2000). Pacing-induced heart failure causes a reduction of delayed rectifier potassium currents along with decreases in calcium and transient outward currents in rabbit ventricle. Cardiovasc Res.

[R15] Gross GJ (2006). Bradycardia-mediated ventricular electrical remodeling. J Cardiovasc Electrophysiol.

[R16] Bozkaya YT (2007). Repolarization characteristics and incidence of Torsades de Pointes in patients with acquired complete atrioventricular block. Anadolu Kardiyol Derg.

[R17] Subbiah RN (2007). Genetically-mediated torsades de pointes during complete heart block. Circulation.

[R18] Epstein AE (2008). American College of Cardiology/American Heart Association Task Force on Practice; American Association for Thoracic Surgery; Society of Thoracic Surgeons. ACC/AHA/HRS 2008  guidelines for Device-Based Therapy of Cardiac Rhythm Abnormalities: executive summary. Heart Rhythm.

